# Supplementary material for: A phosphorylation switch turns a positive regulator of phototropism into an inhibitor of the process
Source: Nat Commun. 2018 Jun 19;9:2403. doi: 10.1038/s41467-018-04752-1 (PMC6008296; doi:10.1038/s41467-018-04752-1)
Supplement: Supplementary file 1 — Supplementary Information [file 41467_2018_4752_MOESM1_ESM.pdf]

Supplementary Information for "A phosphorylation switch turns a positive regulator of phototropism into an inhibitor of the process" by Paolo Schumacher, Emilie Demarsy, Patrice Waridel, Laure Allenbach Petrolati, Martine Trevisan and Christian Fankhauser.

Consists of 6 supplementary figures.

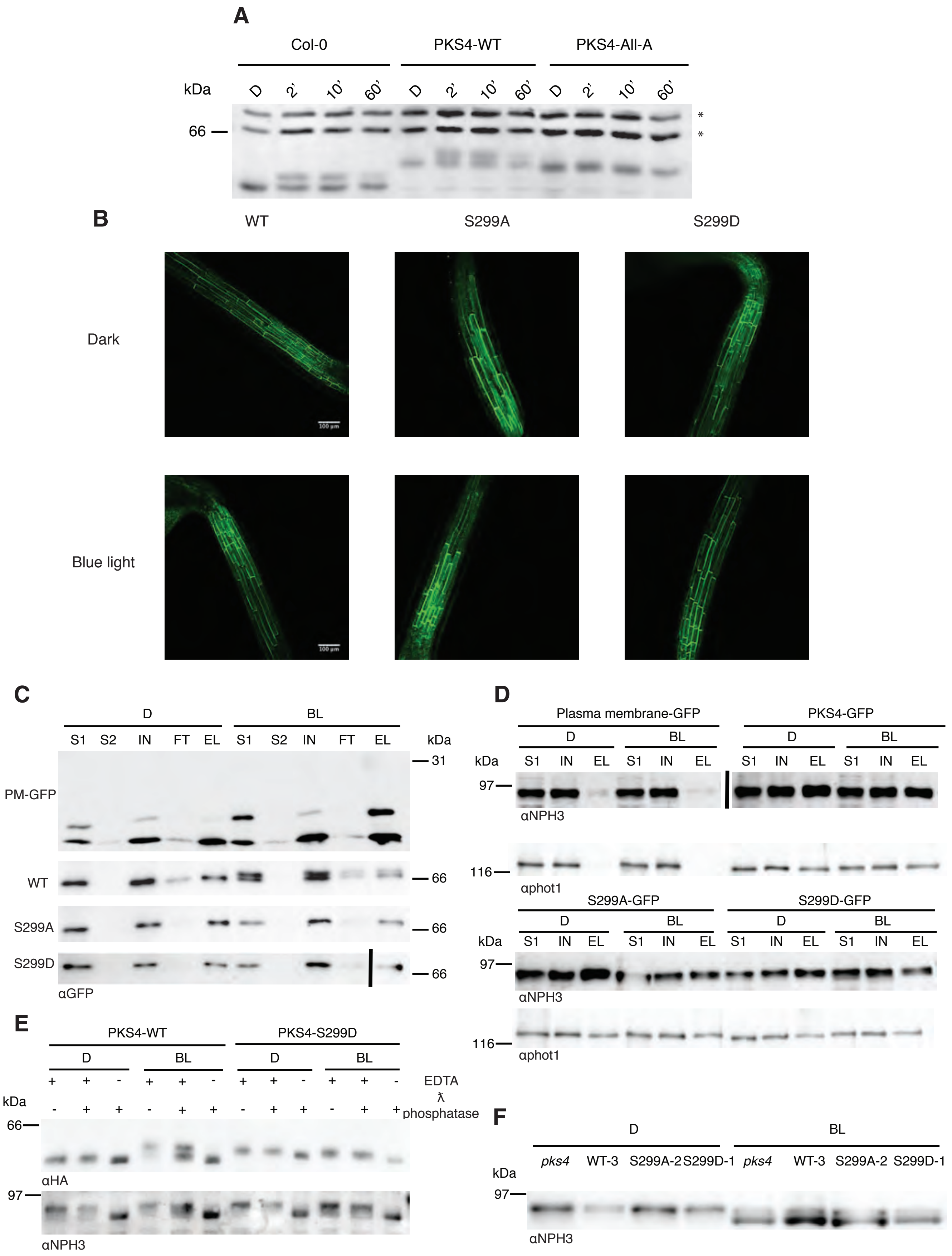

Supplementary Figure 1. PKS4-S299 mutations do not affect protein biochemical properties. Full legend on the next page

Supplementary Figure 1. PKS4-S299 mutations do not affect protein biochemical properties.

(A)(C)(D)(E)(F) 3-day-old dark-grown seedlings protein samples were separated by SDS-PAGE and transferred onto a nitrocellulose membrane. (A) Seedlings were exposed to 15 μmol/m^2/s of blue light for 2, 10 or 60 minutes. Protein accumulation was analyzed by immunobloting using anti-PKS4 antibody. Accumulation of PKS4L in Col-0 and PKS4 WT line is transient. No appearance of PKS4L in PKS4-All-A line at any time point. Asterisks indicate unspecific bands. (B) 3-day-old dark-grown seedlings were kept in the dark or treated with 20 μmol/m^2/s of blue light for 10 minutes. Confocal laser-scanning microscopic (Zeiss LSM 710 NLO) images from *pks4-2* plants containing a PKS4::PKS4::GFP construct (PKS4 CDS either WT, S299->A or S299->D). All versions of PKS4 localize at the periphery of the cell, both in dark and light conditions. (C)(D) 3-day-old dark-grown seedlings were kept in the dark or treated with 100 μmol/m^2/s of blue light for 1 minute. Microsomal protein extracts were co-immunoprecipitated using an anti-GFP antibody. Proteins were separated by SDS-PAGE and transferred onto a nitrocellulose membrane. S1 corresponds to total protein extract, S2 to soluble protein extract, IN to microsomal protein extract used as input for co-immunoprecipitation, FT to flow-through of the column and EL as IP eluate. (C) Protein accumulation was analyzed by immunobloting using anti-GFP antibody. All versions of PKS4 are found in the microsome extract, consistent with membrane association. Both PKS4D and PKS4L are found in the IP eluate. (D) Protein accumulation was analyzed by immunobloting using anti-phot1 and anti-NPH3 antibodies. phot1 and NPH3 are detectable in co-immunoprecipitated extracts from PKS4-WT, S299A and S299D but not in the plasma membrane-GFP line. (E) 3-day-old dark-grown seedlings were either kept in the dark or treated with 150 μmol/m^2/s of blue light for 30 seconds. Proteins were extracted in RIPA buffer and treated with: λ phosphatase alone, λ phosphatase and EDTA or EDTA alone. NPH3 serves as a control for dephosphorylation. (F) 3-day-old dark-grown seedlings were exposed to 15 μmol/m^2/s of blue light for 10 minutes. NPH3 accumulation was analyzed by immunobloting using an anti-NPH3 antibody. Blue-light dependent de-phosphorylation of NPH3 is present in all lines analyzed.

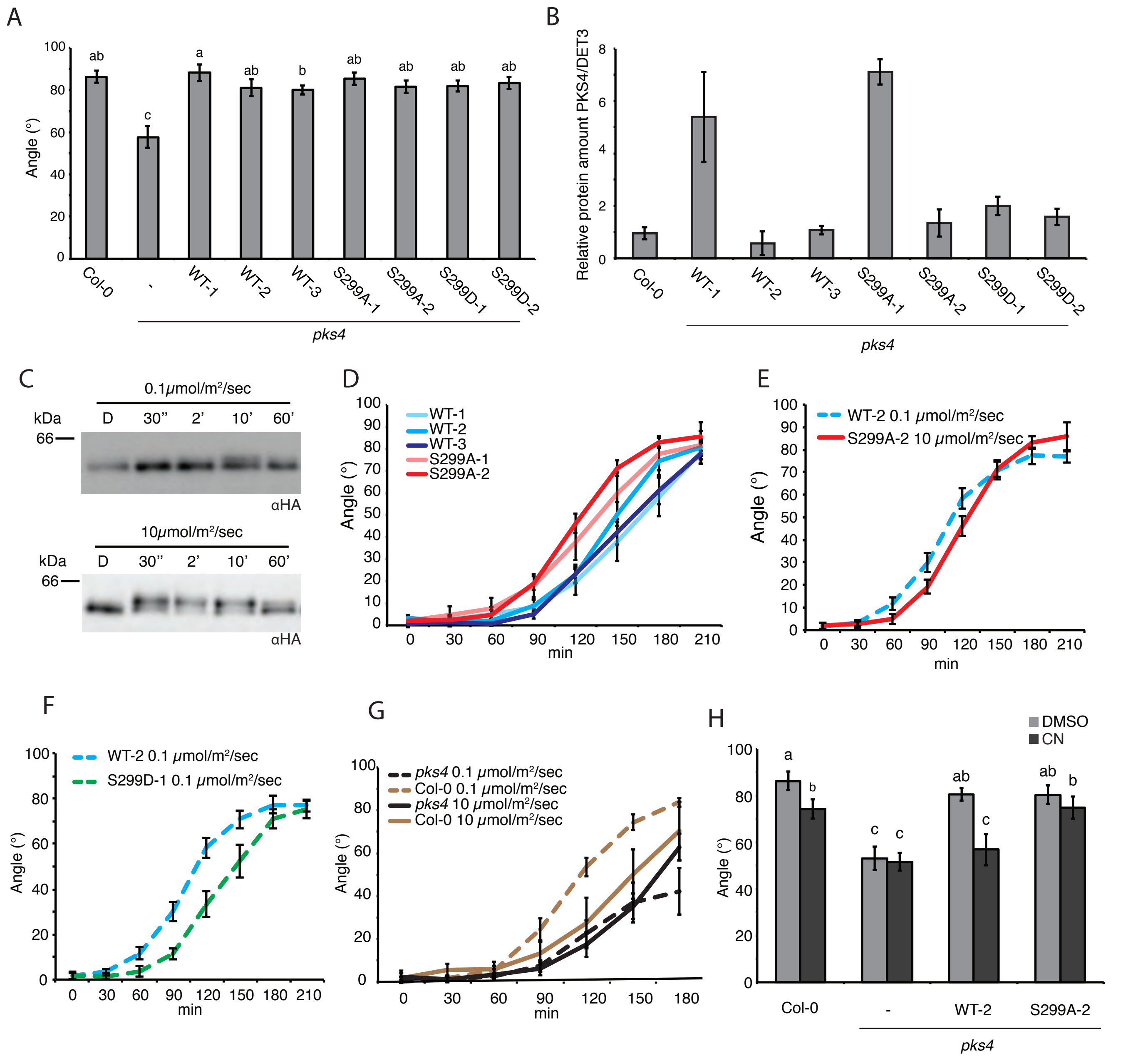

Supplementary Figure 2: PKS4L acts as a negative regulator of phototropism.
(A) 3-day-old dark-grown seedlings were treated with 0.1 µmol/m2/s of lateral blue light for 24 hours. All PKS4-HA lines complement *pks4*. N = 27 to 48. (B) 3-day-old dark grown seedlings protein samples were separated by SDS PAGE and transferred onto nitrocellulose membrane. Quantifications of PKS4 relative to DET3 levels were performed on three biological replicates and 2 technical replicates. (C) 3-day-old dark-grown *pks4* PKS4-WT3 seedlings were exposed to 0.1 or 10 µmol/m2/s of blue light for 0, 0.5, 2, 10 or 60 minutes. Proteins were separated by SDS-PAGE and transferred onto nitrocellulose membrane. PKS4 accumulation was analyzed by immunobloting using anti-HA antibodies. Accumulation of PKS4L is barely detectable at 0.1 µmol/m2/s but clear at 10 µmol/m2/s. (D) 3 independent single insertion lines of *pks4* PKS4-WT and 2 of *pks4* PKS4-S299A were treated with or 10 µmol/m2/s of lateral blue light for the indicated time. Single insertion lines from the same construct have similar phenotypes. Data for *pks4* WT-2 and *pks4* S299A-2 are replotted from Fig. 2 A and B. For *pks4* WT-1, data from 2 independent experiments are represented N = 13 to 23. For *pks4* WT-3, data from 2 independent experiments are represented N = 12 to 30. For *pks4* S299A-1, data from 1 experiments are represented N = 8 to 15. (E) Data shown in Fig. 2 B and C were pooled and replotted. 3-day-old dark-grown seedlings were treated with 0.1 or 10 µmol/m2/s of lateral blue light for the indicated time. *pks4* PKS4-S299A-2 at high light intensity has a similar response than *pks4* PKS4-WT-2 at low light intensity. (F) 3-day-old dark-grown seedlings were treated with 0.1 µmol/m2/s of lateral blue light for the indicated time. *pks4* S299D-1 has a reduced phototropic response compared to *pks4* WT-2. Data for pks4 WT-2 are replotted from Fig. 2 A. For *pks4* S299D-1, data from 2 independent experiments are represented N = 18 to 25. (G) 3-day-old dark-grown Col-0 and *pks4* seedlings were treated with or 10 µmol/m2/s of lateral blue light for the indicated time. *pks4* is impaired at 0.1 µmol/m2/s but not at 10 µmol/m2/s. For 0.1 µmol/m2/s, data from 2 independent experiments are represented N = 16 to 36. For 10 µmol/m2/s, data from 1 experiments are represented N = 5 to 11. (H) 3-day-old dark-grown seedlings were treated with 0.1 µmol/m2/s of lateral blue light for 7 hours. Seedlings were transferred into new plates containing 10 µmol CN 2 hours prior to the light treatment. *pks4* PKS4-S299A-2 is not affect by CN treatment. N = 17 to 26. (A)(D)(E)(F)(G)(H) Angle of curvature was measured as the angle of deviation from the vertical. Error bars represent 2x standard error. Means with same letter are not significantly different (p>0.05, ANOVA tests, one-way for panel A and two-way for panel H). (D)(E)(F)(G)(H) Only seedlings with the apical hook facing towards the light were measured.

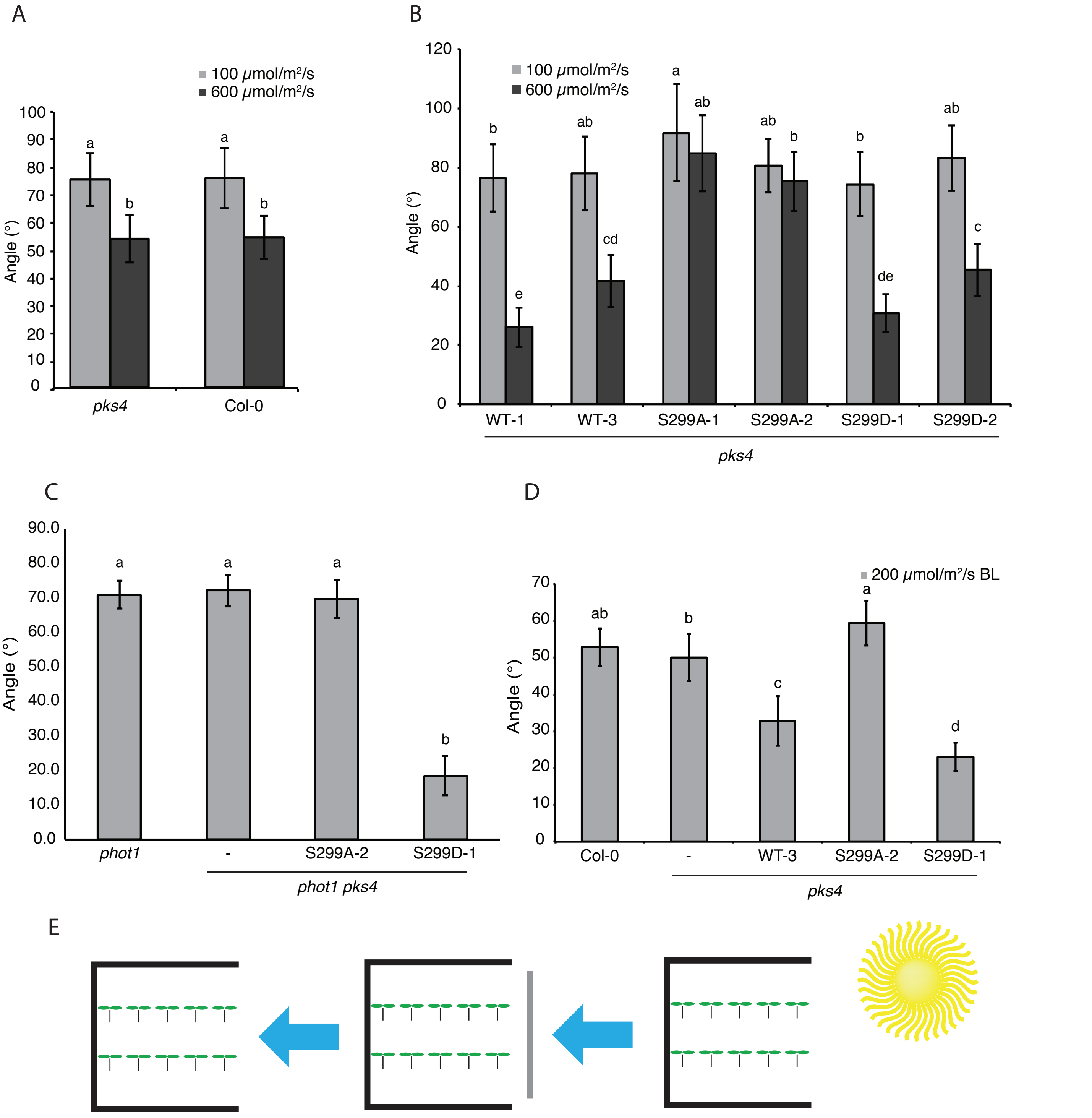

Supplementary Figure 3: High light reduces phototropism through PKS4L accumulation in light grown seedlings.
(A) Col-0 and *pks4* Seedlings grown for 3 days in a 16h-8h day-night cycle were treated with 100 or 600 µmol/m2/s of unilateral blue light for 6 hours. The experiment started 2 hours after dawn (ZT2). Both Col-0 and *pks4* show a reduced phototropic response at 600 µmol/m2/s. N = 49 to 56 (B) Seedlings grown for 3 days in a 16h-8h day-night cycle were treated with 100 or 600 µmol/m2/s of unilateral blue light for 6 hours. The experiment started 2 hours after dawn (ZT2). 2 independent insertion lines from *pks4* PKS4-WT, *pks4* S299A and *pks4* S299D were tested and showed similar results within genotypes. N = 34 to 61. (C) Seedlings grown for 3 days in a 16h-8h day-night cycle were treated with 600 µmol/m2/s of unilateral blue light for 6 hours. Note the strongly reduced phototropic response of *phot1 pks4* S299D-1 while the other genotypes have a similar response (D) Seedlings were grown for 3 days in a 16h-8h day-night cycle. Seedlings were placed in a greenhouse for 3 hour and 30minutes. Blue light was ~200 µmol/m2/s and red light ~600 µmol/m2/s. The experiment started 4 hours after dawn (ZT4). pks4 S299A-2 shows a stronger phototropic response than the others lines. N = 59 to 84. (A)(B)(C)(D) Angle of curvature was measured as the angle of deviation from the vertical. Error bars represent 2x standard error. Means with same letter are not significantly different ($p>0.05$, ANOVA test, two way for panels A and B, one way for panels C and D). (E) Graphic representation of experimental set-up for unilateral exposure phototropism experiments. Plants sown in plates are placed in a black box open only on one side. Blue arrow represents monochromatic blue light. The grey line represents a neutral density filter used to reduce total light intensity. The same set-up was used for experiments under monochromatic light using LED light or for greenhouse experiments with exposure to direct sunlight.

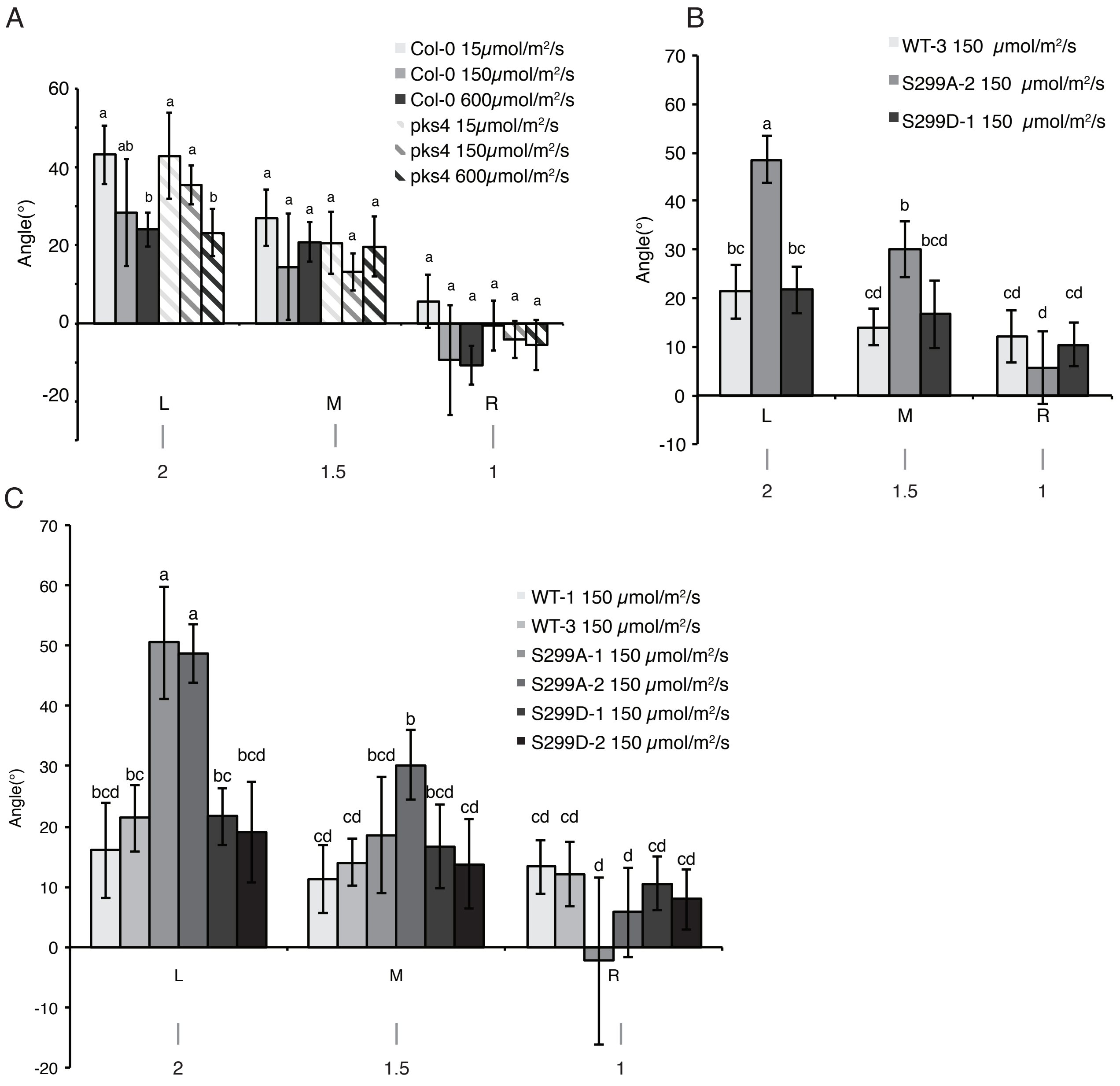

Supplementary Figure 4: Phototropic response to light gradient of Col-0, *pks4* and PKS4-S299D. Phototropic bending in bilateral illumination experiments performed as in Figure 4. The light ratio on the three positions on a plate is indicated below. Positive values correspond to bending towards the left of the plate, negative values correspond to bending towards the right. Data represent means +/- 2x standard error. Means with same letter are not significantly different ($p>0.05$, ANOVA tests, one-way test for each position panel A, two way for panels B and C). (A) Col-0 and *pks4* at the indicated total fluences. n = 47 to 49. Statistical analysis was performed independently for L, M and R positions to limit the number of factors analyzed. (B) For *pks4* WT-3, *pks4* S299A-2 and *pks4* S299D-1 at a total fluence of 150 μmol m-2s-1 blue light. n = 24 to 28. (C) For *pks4* (WT1 and WT-3), *pks4* (S299A-1 and S299A-2) and *pks4* (S299D-1 and S299D-2) at a total fluence of 150 μmol m-2s-1 blue light. n = 20 to 31.

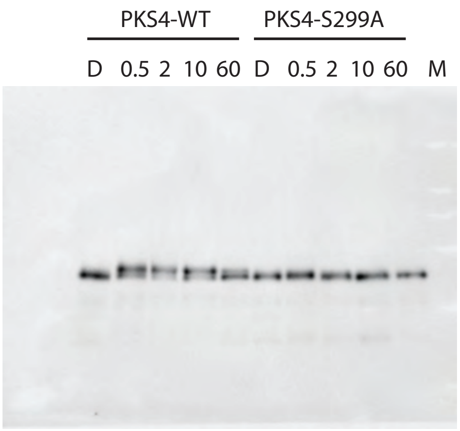

Figure 1C

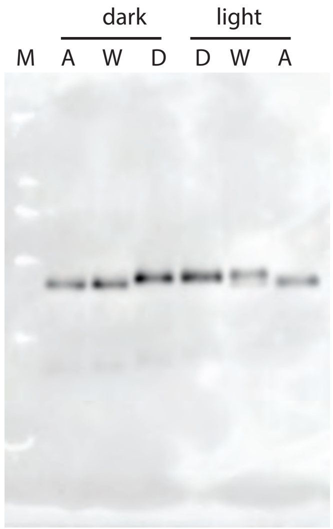

D = S299D
A = S299A
W = WT PKS4

Figure 1D

Supplementary Figure 5: Full western blots of Figure 1

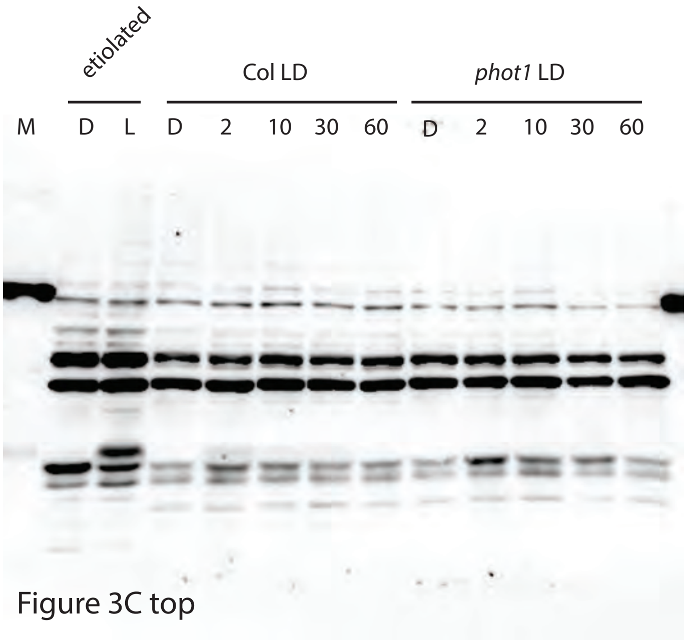

Figure 3C top

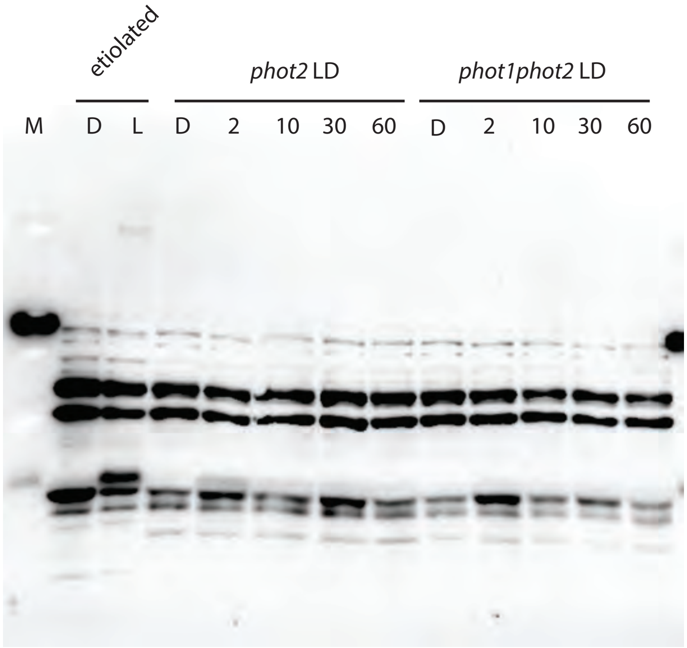

Figure 3C bottom

Supplementary Figure 6: full westerns from Figure 3
